# Molecular detection of *Bordetella holmesii* in two infants with pertussis-like syndrome: the first report from Iran

**Published:** 2017-08

**Authors:** Masoumeh Nakhost Lotfi, Vajiheh Sadat Nikbin, Omid Nasiri, Farzad Badmasti, Fereshteh Shahcheraghi

**Affiliations:** Department of Bacteriology, Microbiology Research Center, Pertussis Reference Lab, Pasteur Institute of Iran, Tehran, Iran

**Keywords:** *Bordetella holmesii*, Real-time PCR, *HIS1001*, Pertussis-like Infections

## Abstract

**Background and Objectives:**

*Bordetella holmesii* is associated with a pertussis-like respiratory syndrome in healthy individuals and also a rare cause of septicaemia, endocarditis, pneumonia, and septic arthritis, mostly in immunocompromised patients. Culture technique and real-time PCR are 2 methods used to detect *Bordetella* spp.

**Materials and Methods:**

In this study, 435 nasopharyngeal specimens of patients with suspected whooping cough were checked for the presence of *B. holmesii* using 2 methods of culture technique and real-time PCR.

**Results:**

In this study, we detected *hIS1001* and *IS481* of *B. holmesii* in 2 infants suspected of having pertussis-like syndrome.

**Conclusion:**

Our observations demonstrate that accurate diagnosis is needed to discriminate between *B. holmesii* and *B. pertussis* infections among pertussis cases; otherwise, it could lead to misestimating pertussis rate and vaccine efficacy.

## INTRODUCTION

*Bordetella holmesii* is a Gram- negative coccobacillus, strictly aerobic and slow-growing organism, which was first identified as a species in 1995 (1). Little information is available on the epidemiology and clinical manifestations of *B. holmesii*. Although *B. holmesii* has not been historically associated with a cough illness, this pathogen has recently been more associated with a pertussis-like respiratory syndrome in healthy individuals (2, 3). It is difficult to ensure whether *B. holmesii* is in fact responsible for the clinical symptoms, as the sequencing of *B. holmesii* genome has harbored any gene encoding virulence factors similar to those of *B. pertussis* (4). Moreover, *B. holmesii* is also a rare cause of septicemia, endocarditis, pneumonia and septic arthritis, mostly in immunocompromised patients (5).

Respiratory infection associated with *B. holmesii* is frequently misidentified as whooping cough with *B. pertussis*. Although no fatal cases of *B. holmesii* have been reported, invasive infections associated with this bacterium can cause substantial morbidities even in previously healthy individuals. Antimicrobial treatment can be difficult because susceptibility of *B. holmesii* to macrolides and third generation cephalosporin is lower than expected (6, 7).

Based on previous investigations, *B. holmesii* does not have a clear reservoir and transmission pattern. It is still unknown whether these species are pathogens for humans or they should be considered as opportunistic bacteria. However, the biological diagnosis has confirmed the presence of *B. holmesii* in human respiratory samples (7–10). Although *Bordetella* spp. are genetically similar to each other, they are different in the presence or absence of insertion sequences (ISs) and their copy number. For example, *IS481* presents in *B. pertussis*, *B. holmesii* and *B. bronchiseptica* with 50–200, 8–10 and <5 copy number, respectively, while it has not been reported in *B. parapertussis*. Moreover, there is *IS1001* in *B. parapertussis* and some of *B. bronchiseptica*; and *hIS1001* has just been found in *B. holmesii* (11). Detection of ISs is the basis of molecular diagnosis among *Bordetella* species. Therefore, correct detection of *Bordetella* spp. need a precise molecular technique such as real-time PCR. On the other hand, the incorrect reports of pertussis-like syndrome associated with *B. holmesii* as whooping cough give rise to misestimating pertussis infection rate. It seems that *B. holmesii* may need a diagnostic assay and epidemiological surveillance. The present study aimed at detecting *B. holmesii* in clinical samples of patients suspected of having pertussis using real time PCR.

## MATERIALS AND METHODS

### Patients, sampling and culture.

Nasopharyngeal samples were taken from 435 pertussis suspected patients in all age ranges using Dacron swab. The swabs were put in Regan-Lowe (RL) agar (with oxacillin) as transport medium. One of the swabs was cultured on RL agar plates (with or without oxacillin in medium) and incubated at 35–36°C for 14 days. The second swab was used for DNA extraction and real-time PCR. In this study, the samples were not checked for the presence of viral or mycoplasmal infections.

### DNA Extraction and Real-time PCR.

DNA was extracted from 200 μL of diluted specimen using High Pure PCR Template Preparation Kit, as recommended by the supplier (Roche Diagnostics, GmbH, Mannheim, Germany). The extracted DNAs were eluted in a 100μL volume. Primers and TaqMan probes were designed to amplify *IS481*, *IS1001*, *IS1002*, *ptxP* and *hIS1001*, as demonstrated in Table 1. ABI 7500 Real-Time PCR System (Applied Bio-system, Inc.) was used for amplification. The temperature profile included an initial denaturation of 10 minutes at 95°C, followed by 45 cycles 95°C for 15 seconds and 60°C for 1 minute. Acquisition of the fluorescence signal was set at 60°C during each cycle. Cycle threshold (Ct) values were determined automatically using the ABI SDS software. Positive control samples of purified DNA from the reference strains were *B. petussis* Tohama I, *B. parapertussis* 12822 and *B. holmesii* ATCC 51541; moreover, no template control samples (PCR-grade water) were included in each run. Eukaryotic *RnaseP* was used as internal control in each tube.

**Table 1.** The primers and TaqMan probes was used in this study

**Primer and probe name**	**Sequence (5′→3′)**	**Final concentration (μM)**	**Reference**
*IS481* Fwd	GCCGGATGAACACCCATAAG	0.25	(20)
*IS481* Rev	GCGATCAATTGCTGGACCAT	0.25	
*IS481* probe^(FAM)^	CGATTGACCTTCCTACGTC-MGB	0.1	
*IS1001* Fwd	AATTGCTGCAAGCCAACCA	0. 25	(20)
*IS1001* Rev	CCAGAGCCGTTTGAGTTCGT	0. 25	
*IS1001* probe^(VIC)^	ACATAGACCGTCAGCAG- MGB	0.1	
*IS1002* Fwd	CTAGGTCGAGCCCTTCTTGTTAAC	0. 25	(20)
*IS1002* Rev	GCGGGCAAGCCACTTGTA	0. 25	
*IS1002* probe^(CY5)^	CATCGTCCAGTTCTGTTGCATCACCC-BHQ-3	0.1	
*ptxP* Fwd	TTCGTCGTACAAAACCCTCGA	0. 25	(21)
*ptxP* Rev	GTTCATGCCGTGTTGGATTG	0. 25	
*ptxP* probe^(FAM)^	CTTCCGTACATCCC-BHQ-1	0.1	
*hIS1001* Fwd	CCGTGCCAATCGGTAAAGTT	0. 25	(22)
*hIS1001* Rev	AAGGGCTGGTTGGCCTGGAGCA	0. 25	
*hIS1001* probe^(FAM)^	GTCCTGCGTGACGAACTCAA	0.1	
*RNaseP* Fwd	CCAAGTGTGAGGGCTGAAAAG	0. 25	(23)
*RNaseP* Rev	TGTTGTGGCTGATGAACTATAAAAGG	0. 25	
*RNaseP* probe^(Yakimayellow)^	CCCCAGTCTCTGTCACCACTCCC-BHQ-1	0.1	

## RESULTS

### Culture and real-time PCR.

The suspensions of samples were cultured on RL agar for 14 days. However, no colonies were isolated as *Bordetella* spp. on the RL agar. Considering the high sensitivity of real-time PCR technique, all 435 samples were checked for *IS481*, *IS1001*, *ptxP* and *hIS1001*. Among the samples, 2 were detected as *B. holmesii*, which had *hIS1001* with Ct value of 37.9 and 37.2, respectively, and also had *IS481* with Ct value of 42.4 and 41.4; however, they were negative for *IS1001* and *ptxP.* The results of culture and real-time PCR of the 2 positive samples are demonstrated in Table 2.

**Table 2.** Laboratory findings for two patients with pertussis-like syndrome

**Patient No.**	**Culture**	***IS481***	***IS1001***	***IS1002***	***ptxP***	***hIS1001***	RnaseP^*^
1	-	+ (41.4)	− (UD)	− (UD)	− (UD)	+ (37.9)	+ (28.83)
2	-	+ (42. 4)	− (UD)	− (UD)	− (UD)	+ (39.2)	+ (30.04)

UD means “undetectable”

* Eukaryotic *RnaseP* was used as internal control in each tube

### Demographic and clinical characteristics of the patients.

The first case belonged to a 5- month-old female, who was admitted to a general hospital in southwest of Iran (Ahwaz, Khuzestan) in July 2015. Her symptoms were proximal cough and post-tussive vomiting but no fever. This patient had received 2 DTP vaccine doses including whole- cell of pertussis (wP) at 2 and 4 month olds. Prior to hospital admission, she had been injected 2 doses of azithromycin (10 mg/kg per day) by a physician. The second case was a 3- year- old female, who referred to a general hospital in northeast of Iran (Shirvan, khorasan) in June 2015. She had received all the vaccines (She had been injected 4 doses at 2, 4, 6, and 18 month olds.). There was no antibiotic prescription for this patient before sampling. Neither of the patients had splenectomy experience or immunocompromised status. However, we found that the two patients had close contacts with persons that had persistent cough.

## DISCUSSION

*Bordetella* spp can be diagnosed using 2 methods: culture and real-time PCR (12). Cephalexin is widely used in culture medium of *Bordetella* spp. (eg, Regan–Lowe agar) and it has been acknowledged that it has an inhibitory effect on the growth of *B. holmesii*, which possibly explains why most laboratories did not identify *B. holmesii* in nasopharyngeal specimens of patients with pertussis-like symptoms before 2000 (13). Hence, meticillin or oxacillin is preferred to be to add to culture medium instead of cephalexin (14). In this study, no colonies were isolated as *B. holmesii* on the RL agar (with or without antibiotic in medium). The most difficulties in the isolation of *Bordetella* spp. are inappropriate techniques of specimen collection, transportation, and detection. Real-time PCR is a sensitive and acceptable assay to detect *Bordetella* spp. infection. Most of the PCR tests are based on detection of insertion sequences (IS) present in multiple copies per genome, increasing the sensitivity of PCR tests. The *IS481* is the target mostly used to detect *B. pertussis*. However, the *IS481*, which has been associated with pertussis-like disease, is also present in *B. holmesii* (15, 16). There are only 2 specific molecular diagnostic approaches available: One for *B. pertussis*, the target is the sequence of the pertussis toxin gene promoter (17) and the other for *B. holmesii*, the target is the *hIS1001* sequence (18). A retrospective study of 177 samples using real-time PCRs specific for *B. pertussis* and for *B. holmesii* showed that *B. holmesii* DNA was detected in 20.3% of the samples collected from adolescents and adults (8, 9).

The pertussis vaccination program in Iran includes 3 doses of a whole-cell pertussis vaccine together with diphtheria and tetanus toxoid (DTwP) at months 2, 4, and 6 of life and 2 booster doses at 18 months and to 6 years old. The incidence of *B. pertussis* in Iran was 0.5 cases per 100 000 population in 2008, which was higher than the previous year, 0.19 cases per 100 000 population (19). We believe that the re-emergence of pertussis is overestimated because the infection associated with *B. parapertussis* and *B. holmesii* are similar to pertussis infection and have usually been reported as pertussis cases, though they have milder nature and shorter duration. Therefore, accurate diagnosis is the utmost importance in evaluating vaccine efficacy. Interestingly, the mice model has shown that neither whole-cell (wP) cellular (aP) *B. pertussis* vaccination conferred protection against *B. holmesii*. Although T-cell responses induced by wP or aP cross-reacted with *B. holmesii*, vaccine-induced antibodies failed to efficiently bind to *B. holmesii* (20).

The reservoir of *B. holmesii* and transmission between humans are currently unknown. However, one report in Japan has demonstrated that 5 patients infected with *B. holmesii* showed epidemiologic linkage (2). In particular, the fact that 4 of these patients attended the same junior high school suggests that *B. holmesii* may be transmitted from person to person (2). However, in their conclusions, the authors explained that they did not check for any other causes of clinical symptoms, such as viral or mycoplasma causes. We have reported the first molecular diagnosis of *B. holmesii* in Iran using real-time PCR. Our observations revealed that accurate diagnosis is needed to discriminate between *B. holmesii* and *B. pertussis* infections among pertussis cases because symptoms associated with these 2 diseases are similar. The laboratory capacity limitations to detect *B. holmesii* from *B. pertussis*, give raise to the rate of infections associated with *B. holmesii*, which have been underestimated. Further studies, surveillance enhancement, and precise diagnosis are required to fully elucidate the burden of *B. holmesii* infections among infants, adolescents and adults.
